# Labor Dystocia as First Presentation of Pelvic Malignancy

**DOI:** 10.1155/2011/584184

**Published:** 2011-07-13

**Authors:** Dennis van Hamont, Petra L. M. Zusterzeel

**Affiliations:** Department of Obstetrics and Gynecology, Radboud University Nijmegen Medical Centre, P.O. Box 9101, 6500 HB Nijmegen, The Netherlands

## Abstract

The underlying causes of labor dystocia can be various. Lack of expulsive forces or fetal malpresentation are amongst the most common ones. However, pelvic masses are described as well. Here we describe two cases of labor dystocia as first presentation of pelvic malignancy.

## 1. Introduction

Labor dystocia is responsible for more than 50 and 20 percent of secondary cesarean sections in nulliparous women and repeat cesareans, respectively [1]. Dystocia is considered the result of (1) abnormalities of expulsive force; (2) abnormalities of presentation, position, or development of the fetus; (3) abnormalities of the maternal pelvis or birth canal; or a combination of these factors [2]. Although infrequently described, pelvic or intra-abdominal masses should be contemplated as an underlying cause of obstructive delivery and should be thought of especially after a normal vaginal delivery in the past. We report two cases of labor dystocia as first presentation of underlying pelvic malignancy.

**Case 1**

After an uncomplicated pregnancy a 32-year-old G2P1 underwent a secondary cesarean section at GA 41 weeks for labor dystocia. Despite active support of labor maximal cervical dilation measured 7 cm, her peripartum medical record reported no abnormal findings. A healthy daughter 3, 170 g was born. During surgery a large elastic to solid abnormality was palpable in the anterior vaginal wall; for no obvious reasons histology samples were not taken.

Her first pregnancy ended in a primary cesarean section for breech presentation and preeclampsia at 37 wks—giving birth to a healthy son 2, 130 g. Further medical history showed an appendectomy and a correction for inguinal herniation.

Four weeks after delivery patient was evaluated at an outpatient clinic of a general nonteaching hospital for vaginal discharge and fever. Again the mass in the anterior vaginal wall was palpable. Vaginal cytological and histological samples were taken but showed no evidence of abnormality. The patient was treated with oral antibiotics (amoxicillin with clavulanic acid) and reassessed 15 days later. At this last visit the mass was still present but its consistency felt rigid. Seven weeks postoperative a MRI-scan of the pelvis showed a tumor 3.5 × 10 cm in the anterior vagina wall with benign impression without any pathological lymph nodes detectable.

Thereafter the patient was referred to our Gynecologic Oncology Department of the Radboud University Nijmegen Medical Centre (RUNMC), Nijmegen, The Netherlands. Here she underwent an examination under anesthesia, combined with a cystoscopy and obtainment of histology samples. A rigid solid tumor was palpated in the anterior vaginal wall reaching from the cervix to midurethral extending laterally to the bilateral parametria. The trigone of the urinary bladder was elevated. Since we suspected ureter obstruction, ultrasonic evaluation of the kidneys followed, showing a dilated left kidney and ureter. A true cut biopsy of the anterior vaginal wall was taken.

Histology of this biopsy revealed a diffuse large B-cell non-Hodgkin lymphoma.

The left kidney function was restored by placing a nephrostomy catheter. Patient was referred to the hematology department for further evaluation and chemotherapy treatment (R-CHOP; Rituximab, Cyclophosphamide, Hydroxydaunorubicin (adriamycin), Oncovin (vincristine), Prednisone). She showed a complete remission after eight R-CHOP courses; also true cut biopsy revealed no evidence of disease. However, since a reduced mass was still visible on CT-scan adjuvant brachytherapy was initiated.

**Case 2**

A second patient was a 38-year-old G6P2 who underwent a secondary cesarean section which was performed for labor dystocia at GA 41 weeks. Her medical history revealed a secondary cesarean for fetal distress and a vaginal delivery. Both children were full-term and healthy.

The pregnancy was complicated by recurrent vaginal blood loss in the second half, without any macroscopic cervical abnormalities seen at gynecologic examination. Cervical cytology at 27-week gestational age showed atypical squamous cells of undetermined significance, and the smear was scheduled to be repeated 6 weeks after delivery. The current delivery was induced for oligohydramnios. The cervix dilated to a maximum of 4 cm, despite good contractions. The cervix felt firm to hard in consistency. After the cesarean section histology samples of the cervix were taken in the same theatre session, showing grade 2 squamous cell carcinoma (SCC). She delivered a healthy son, 4,084 g.

After the diagnosis of SCC of the cervix, she was referred to our Gynecologic Oncology Department (RUNMC) and underwent an examination under anesthesia, revealing a severely altered cervix with a tumor measuring 5 cm involving the right parametrium. She was clinically staged as a cervical cancer FIGO stage IIb. CT-scan showed no pathological lymph nodes, but due to the short postpartum interval the uterine cervix could not be assessed reliably.

Since delivery by cesarean section might have led to spill of tumor cells intra-abdominally or might have provoked hematological metastasis, the patient received 6 courses neoadjuvant chemotherapy consisting of cisplatinum and placitaxel, both 70 mg/m^2^. Examination under anesthesia after chemotherapy showed a substantial decrease in tumor size to 1.5 cm. Patient was scheduled for radical hysterectomy, bilateral salpingo-ooferectomy, and bilateral pelvic lymphadenectomy. Histopathological analysis of the specimens showed no further malignancy; all 18 removed pelvic lymph nodes were negative for malignancy.

## 2. Discussion

Cervicovaginal, intraabdominal, or retroperitoneal abnormalities obstructing labor have been described since one of the first publications by Freeth in 1950 [3]. These abnormalities can be both benign [4–6] or malignant [7–9]. 

In the Netherlands cancer is among the leading causes of nonaccidental death in young women, with mortality rates of 26% in women aged 20 to 30 years and 42.5% aged 30 to 40 years [10]. Therefore, it is not surprising that an estimated 1 in 1000 women is affected by cancer in pregnancy [11]. This rate may further increase due to the tendency of postponing pregnancy to the latter reproductive years. 

Gynecologic malignancies associated with pregnancy can be detected and diagnosed throughout pregnancy. However, most gynecological cancers are found in the first and second trimester of gravidity. Recurrent vaginal blood loss or abnormal findings on prenatal ultrasound screening may lead to early diagnosis. Adequate and repeated pelvic examination throughout routine antenatal care may detect pelvic masses at an early stage. This might reduce obstructed and complicated deliveries, but more importantly it may prevent late complications due to metastatic disease [12]. Early diagnosis and treatment is also important since significantly better survival is observed in patients in whom cancer was detected in early pregnancy [11].

As a result of frequent antenatal examination, obstructive labor is seldom the first presentation of a pelvic malignancy. However, it still occurs, and pelvic malignancy obstructing labor should be considered peripartally in case of dystocia.
